# Editorial: Linking nitrogen cycling transformations to microbial diversity in freshwater ecosystems

**DOI:** 10.3389/fmicb.2022.1098905

**Published:** 2022-11-29

**Authors:** Antonio Castellano-Hinojosa, Jesús González-López, Laura M. Cardenas, Sarah L. Strauss

**Affiliations:** ^1^Southwest Florida Research and Education Center, Department of Soil, Water, and Ecosystem Sciences, Institute of Food and Agricultural Sciences, University of Florida, Immokalee, FL, United States; ^2^Department of Microbiology, Institute of Water Research, University of Granada, Granada, Spain; ^3^Net Zero and Resilient Farming, Rothamsted Research, North Wyke, Devon, United Kingdom

**Keywords:** nitrogen, microbial diversity, aquatic ecosystems, denitrification, greenhouse emissions, freshwater ecosystem

## Introduction

Increases in the availability of reactive nitrogen (Nr) in the environment due to human activities has altered the cycle of nitrogen (N) over the last century (Galloway and Cowling, 2021). Among global ecosystems, freshwater ecosystems (e.g., rivers and lakes) are considered suitable models to examine changes in processes related to the functioning of the N cycle as they are sensitive to nutrient loads across large spatial and temporal scales (Smith, 2003; Catalán et al., 2006; Capon et al., 2021; Medina-Sánchez et al., 2022). In mountain freshwater ecosystems, Nr is mainly introduced *via* wet deposition in the form of nitrate (${}_{3}^{-}$), but human activities can also introduce N into aquatic ecosystems at lower altitudes (Castellano-Hinojosa et al., 2017, 2022; Siles and Margesin, 2017).

Within the N cycle, denitrification is the main biological process that contributes to the removal of Nr species from aquatic ecosystems (Castellano-Hinojosa et al., 2017, 2022; Palacin-Lizarbe et al., 2020). The denitrification process, the sequential reduction of ${\text{NO}}_{3}^{-}$ to dinitrogen (N_2_), is also highly relevant to the biosphere because it is the primary biological process of removing the potent greenhouse gas nitrous oxide (N_2_O) from the atmosphere. However, other processes, such as nitrification, can contribute to reducing Nr in freshwater ecosystems by providing extra ${\text{NO}}_{3}^{-}$ for denitrifiers.

Despite the key role of microbial communities in N-cycling, the linkages between N-transformations and microbial abundance and diversity are largely unknown in freshwater ecosystems. Because N-transformation dynamics in these ecosystems can be episodic and spatially heterogeneous, the study of the linkages between variations in abiotic factors (e.g., ${\text{NO}}_{3}^{-}$, salinity, temperature, and oxygen availability) to microbial communities may help elucidate how N-cycling transformations occur in aquatic ecosystems, how these ecosystems respond to increased inputs of Nr, and whether they may act as an auto-depurative mechanism for removal of Nr. In addition, how alterations of the N cycle in aquatic ecosystems may influence other biogeochemical cycles [e.g., carbon dioxide (CO_2_) and methane (CH_4_) emissions] has been poorly studied.

This Research Topic compiles scientific contributions that increase our understanding of how microbial communities drive changes in N-cycling transformations and greenhouse emissions in different freshwater ecosystems, and how these transformations may be related to alterations in other biogeochemical cycles.

## Microbial community composition in aquatic ecosystems

Understanding the relationships and mechanisms between biodiversity and ecosystem functions is important for predicting aquatic ecosystem responses to global climate changes (Martiny et al., 2011; Wang et al., 2022). The use of beta diversity is a well-known metric to study changes in microbial community composition (Mena and Vázquez-Domínguez, 2005). In freshwater ecosystems, variations in microbial community composition have been examined across temporal, altitudinal, and spatial scales (Wang et al., 2020; Yuan et al., 2021). However, few studies have explored the impact of water depth on beta diversity across taxonomic groups. Yuan et al. examined the mechanisms that underline variations in the composition of bacterial, archaeal, and fungal communities in a semi-arid lake along a water depth gradient using two different beta diversity metrics: species turnover (one species replaces another without changing species richness) and nestedness (richness differences attributable to species gain or loss). The authors found that partitioning beta diversity is an effective way to unravel changes in community composition and that the response mechanism of bacterial, archaeal, and fungal communities varied at different water depths. The composition of bacterial and archaeal communities significantly varied across depths in the lake and these changes were mainly controlled by nitrite (${\text{NO}}_{2}^{-}$) and NO${}_{3}^{-}$ contents. However, variations in fungal beta diversity showed no clear pattern across water depths suggesting they may be controlled by other environmental and/or abiotic factors.

## Environmental processes drive changes in microbial communities in aquatic ecosystems

Deterministic or stochastic processes have been used to explain changes in microbial community composition in aquatic and terrestrial ecosystems (Vellend and Agrawal, 2010; Chase and Myers, 2011). In deterministic processes, abiotic and/or biotic factors determine variations in community composition whereas in stochastic processes, probabilistic dispersal and random dynamics are observed. However, how these two processes can influence N-cycling communities has been poorly explored in aquatic ecosystems such as aquaculture ponds. These ponds are subjected to sediment disturbances due to fish activities and nutrient accumulation during the rearing of aquatic organisms. Dai et al. found significant temporal changes in ammonia-oxidizing archaea (AOA) and ammonia-oxidizing bacteria (AOB) community diversity and abundances in aquaculture ponds sediments across three different regions in China. While no temporal changes in community composition of AOA and AOB were observed, there were site-specific AOA and AOB taxa correlated with environmental factors. The significant correlation between geographic distance and AOA and AOB community composition suggested that dispersal limitation (a stochastic process) rather than abiotic and/or biotic factors contributed to variations in AOA and AOB communities in these aquaculture ponds.

## Denitrifiers are key drivers of nitrogen removal in freshwater ecosystems

Endorheic lakes are the primary available water source in arid regions and contribute to the social and economic development of these regions (Tao et al., 2015). The removal of N in these ecosystems depends mainly on the self-purification capacity of the system (Valiente et al., 2018) because they are land-locked drainage networks (Yapiyev et al., 2017). Increased temperature and salinity in arid endorheic lakes due to climate change is introducing new challenges for these aquatic ecosystems that may impact how Nr is removed (Tao et al., 2015; Greaver et al., 2016; Lin et al., 2017). In a endorheic lake in Northwest China, Jiang et al. found that lake sediments had a high potential for the use of denitrification as a way to efficiently remove N from sediments. In water, low ${\text{NO}}_{3}^{-}$ concentrations and abundance of nitrifiers limited denitrification. Increased salinity was related to decreased abundance and diversity of *nosZ*I-type denitrifiers in sediments, suggesting a lower potential to carry out complete denitrification in sediments of endorheic lakes in these arid regions in the future.

## Linking N-cycling transformations, CH_4_ emissions and microbial communities in wetlands

Tropical and subtropical wetlands are major sources of the greenhouse gas CH_4_ because of their elevated net primary productivity and high anaerobicity and temperatures (Bloom et al., 2012). In these ecosystems, the production and consumption of CH_4_ is carried out by methanogenic and methanotrophic microorganisms, respectively. In wetlands, the balance between production and consumption processes of CH_4_ is controlled by the flood pulse that changes soil water saturation and favors methanogenic or methanotrophic activity (Meyer et al., 2017). Microorganisms such as ammonia oxidizers (AMO) and nitrogen-dependent anaerobic methane oxidizers (N-DAMO) can couple the reduction of ${\text{NO}}_{2}^{-}$ and ${\text{NO}}_{3}^{-}$ with the oxidation of CH_4_ for energy production and effectively link the carbon (C) and N cycles (Haroon et al., 2013). The results of Monteiro et al. showed there exists a close relationship between AMO and N-DAMO communities during the flood season in wetlands, as ammonia oxidation can provide oxidized N forms that can be coupled to anaerobic CH_4_ oxidation. Changes in composition of AMO and N-DAMO communities were determined by seasonal changes in soil water saturation and had significant effects on variations in specific soil properties. Together, results of this study revealed there exists a complex balance between C and N cycles in Amazonian wetlands that appears to be controlled by how different N-cycling microbial communities respond to environmental conditions.

## Outlook and challenges ahead

Wet deposition of Nr, increased anthropogenic N inputs into natural environments, and accelerated climate warming are expected to cause major challenges for N-cycling transformations in freshwater ecosystems in the future (Capon et al., 2021). Although the importance of microbial activity to ecosystem function in aquatic ecosystems has been explored, the identification of abiotic and biotic factors driving changes in N-cycling, N-transformation rates, and N-cycling communities in freshwater ecosystems deserves more attention. In particular, improved characterization of N-functional pathways contributing to the emission of greenhouse gases such as N_2_O and additional studies of the relationships between N-cycling and other biogeochemical cycles are needed. Future studies should also explore the effect of altitudinal gradients and inter-annual variability on N-cycling and microbial communities. Results from this future work will provide critical information needed to continue to address environmental and ecological problems related to excessive N content and increased greenhouse emissions.

## Author contributions

All authors listed have made a substantial, direct, and intellectual contribution to the work and approved it for publication.

## Funding

LC was supported by grant number BBS/E/C/000I0320. AC-H was a recipient of a grant of PAIDI 2020, Junta de Andalucía (POSTDOC_21_00255).

## Conflict of interest

The authors declare that the research was conducted in the absence of any commercial or financial relationships that could be construed as a potential conflict of interest.

## Publisher's note

All claims expressed in this article are solely those of the authors and do not necessarily represent those of their affiliated organizations, or those of the publisher, the editors and the reviewers. Any product that may be evaluated in this article, or claim that may be made by its manufacturer, is not guaranteed or endorsed by the publisher.
